# CTLA4^+^CD4^+^CXCR5^−^FOXP3^+^ T cells associate with unfavorable outcome in patients with chronic HBV infection

**DOI:** 10.1186/s12865-022-00537-w

**Published:** 2023-01-12

**Authors:** Chunhua Wen, Zheyu Dong, Yiyue Wang, Guofu Ye, Yanchen Ma, Xuan Yi, Yang Zhou, Xiaoyi Li, Xinchun Zheng, Jinlin Hou, Yongyin Li, Libo Tang

**Affiliations:** 1 State Key Laboratory of Organ Failure Research, Guangdong Provincial Key Laboratory of Viral Hepatitis Research, Department of Infectious Diseases, Nanfang Hospital, Southern Medical University, No. 1838 North Guangzhou Avenue, Guangzhou, 510515 China; 2 Department of Infectious Diseases, The Fifth Affiliated Hospital of Sun Yat-Sen University, Zhuhai, Guangdong China

**Keywords:** HBV, Antiviral therapy, Unfavorable outcome, CD4^+^CXCR5^−^FOXP3^+^T cells, CTLA4

## Abstract

**Background:**

A major barrier to achieving a favorable outcome of chronic HBV infection is a dysregulated HBV-specific immune response resulting from immunosuppressive features of FOXP3^+^ T cells. A better definition of FOXP3^+^ T cells is essential for improving the prognosis of HBV infection. We aimed to investigate the role of CD4^+^CXCR5^−^FOXP3^+^ T cells with CTLA4 expression in patients with chronic HBV infection.

**Methods:**

Treatment-naïve chronic HBV-infected patients, HBV-related hepatic failure, and a longitudinal cohort of chronic hepatitis B (CHB) patients with nucleos(t)ide analogue treatment were enrolled for analysis of CD4^+^CXCR5^−^FOXP3^+^ T cell responses by flow cytometry and single-cell RNA sequencing (scRNA-seq).

**Results:**

ScRNA-seq revealed that circulating CD4^+^CXCR5^−^FOXP3^+^ T cells presented distinct inhibitory features compared to spleen tissue. Meanwhile, patients with treatment-naïve chronic HBV infection or with HBV-related hepatic failure showed an upregulation of immune-suppressive features (PD-1, CTLA4, GITR) on CD4^+^CXCR5^−^FOXP3^+^T cells; in vitro analysis found HBeAg and HBcAg stimulation induced elevated levels of inhibitory molecules. Notably, the frequency of CTLA4^+^CD4^+^CXCR5^−^FOXP3^+^ T cells was positively correlated with HBV DNA levels, and longitudinal analysis demonstrated a high frequency of this subset at 12 weeks of antiviral treatment predicted unfavorable outcome in CHB patients.

**Conclusions:**

CTLA4^+^CD4^+^CXCR5^−^FOXP3^+^ T cells are related to unfavorable outcomes in HBV-infected patients; these data indicated that alleviating CTLA4^+^CD4^+^CXCR5^−^FOXP3^+^ T cells may improve the prognosis of HBV infection.

**Supplementary Information:**

The online version contains supplementary material available at 10.1186/s12865-022-00537-w.

## Background

Hepatitis B virus (HBV) infection is a global health issue, with approximately 250 million individuals exposed chronically and at a high risk of dying from end-stage liver disease or hepatocellular carcinoma (HCC) [1, 2]. Nucleos(t)ide analogues (NAs), the most common options for anti-HBV therapy [3], were reported to correlate with viral control, normalization of liver enzymes, regression of cirrhosis, and reduction in the risk of HCC development [4, 5]. Nevertheless, current antiviral drugs do not always induce favorable treatment responses in patients with CHB. A major barrier to achieving a favorable outcome of chronic HBV infection is an exhausted and dysregulated HBV-specific immune response resulting from immunosuppressive features [6]. Therefore, alleviating the inhibitory features of the immune cells may improve the prognosis of HBV infection.

The inhibitory features were mainly mediated by FOXP3^+^ regulatory T (Treg) cells, and considerable heterogeneity exists among FOXP3^+^Treg cells. Thymus-derived Treg (tTreg) cells, peripherally derived Treg (pTreg) cells, and in vitro-induced Treg (iTreg) cells can be defined based on their developmental origin [7, 8]; additional subpopulations, including activated (or “effector”) Treg cells and resting (or “central”) Treg cells, display a distinct gene expression profile [9]. Populations of human Treg cells with specific phenotypes or cytokines vary in unique functional capabilities. FOXP3^+^Treg cells with CD39 and CD73 expression can drive the accumulation of adenosine nucleosides, which disrupt effector cell metabolism [7, 10]; IL-10-producing cells were enriched in classical FOXP3^+^ Treg cells induced by HBcAg, while blocking IL-10-producing FOXP3^+^ Treg cells restored the function of IFN-γ-secreting cells [11]. Of note, FOXP3^+^Treg cells express co-inhibitory molecules (such as cytotoxic T lymphocyte antigen 4 (CTLA4) and lymphocyte activation gene 3 protein (LAG3)) and can modulate the activity of antigen-presenting cells [7]. Depleting FOXP3^+^Treg cells or inhibiting their function by CTLA4 blocking restored the HBV-specific follicular helper T (Tfh)-B cells response [6]. Thus, FOXP3^+^ cells with CTLA4 expression might be a potential biomarker associated with anti-HBV treatment response.

Herein, by a series of patients with chronic HBV infection in both cross-sectional treatment-naïve cohorts and longitudinal telbivudine treatment follow-up cohorts, we revealed that circulating CD4^+^CXCR5^−^FOXP3^+^ T cells exhibited an upregulation of immunosuppressive features (programmed death receptor-1 (PD-1), CTLA4, glucocorticoid-induced tumor necrosis factor receptor (GITR)) in patients with treatment-naïve chronic HBV infection or with HBV-related hepatic failure. Notably, circulating CD4^+^CXCR5^−^FOXP3^+^ T cells with CTLA4 expression was related to an unfavorable outcome in CHB patients receiving antiviral treatment, implicating that the CTLA4^+^CD4^+^CXCR5^−^FOXP3^+^ T cells might be a novel immunotherapy target for HBV cure.

## Results

### Circulating CD4^+^CXCR5^−^FOXP3^+^ T cells exhibit unique immune-suppressive features

**Fig. 1:**
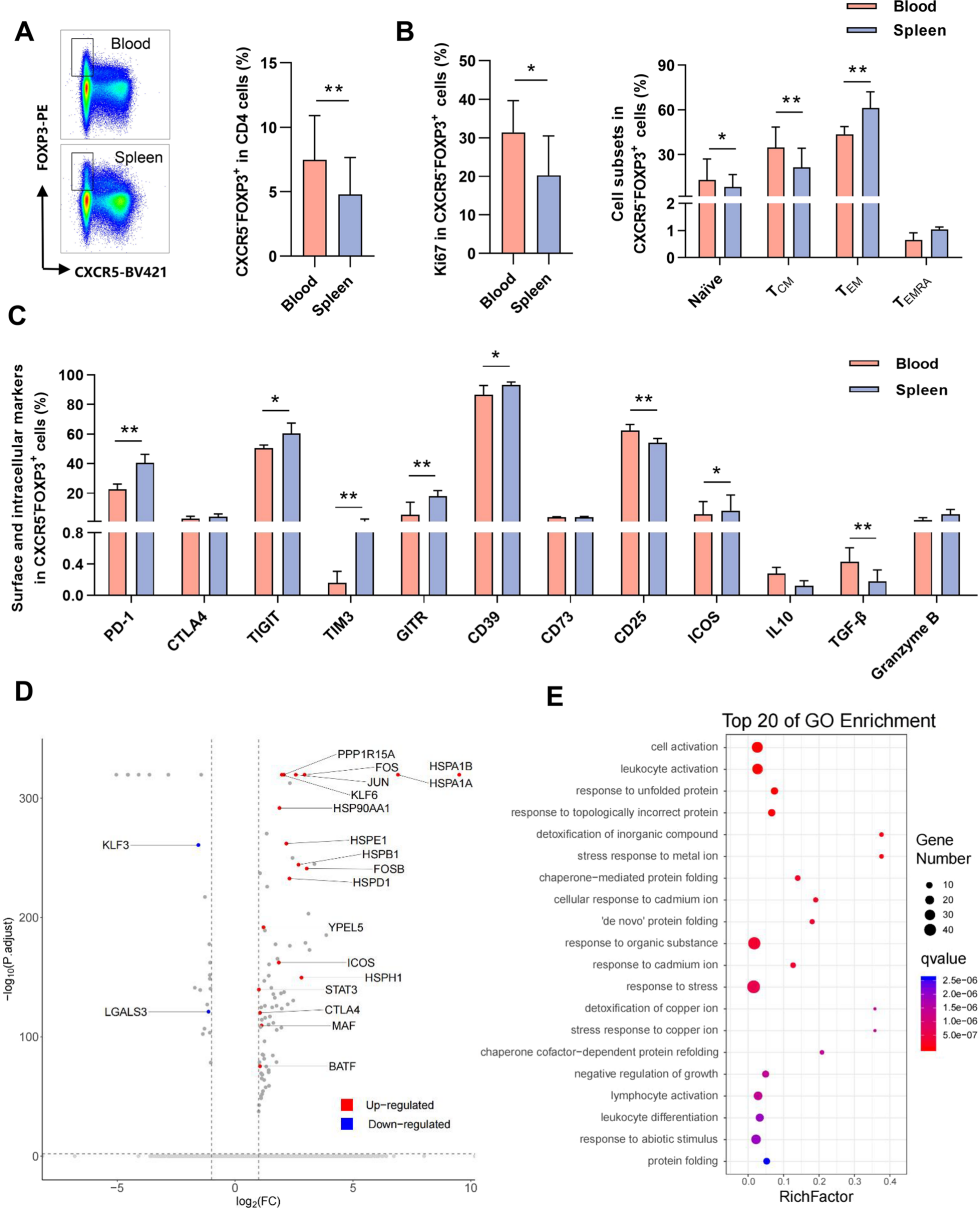
Identification of CD4^+^CXCR5^−^FOXP3^+^ T cells in peripheral blood and spleen. (**A**) The frequency of CD4^+^CXCR5^−^FOXP3^+^ T cells in paired peripheral blood and spleen among patients with HBV infection (n = 9). Horizontal bars reflect the median. (**B**) The percentage of ki67 and T cell subsets (n = 9) in CD4^+^CXCR5^−^FOXP3^+^ T cells among two groups. T cell subsets were defined using the following gating strategy: naïve T cells (CCR7^+^CD45RO^−^), T_CM_: central memory T cells (CCR7^+^CD45RO^+^), T_EM_: effector memory T cells (CCR7^−^CD45RO^+^), T_EMRA_: CD45RA expressing effector memory T cells (CCR7^−^CD45RO^−^) (**C**) The expression of surface and intracellular cytokines (n = 9) in CD4^+^CXCR5^−^FOXP3^+^ T cells among the above groups. (**D**) Volcano plot of differentially expressed genes in circulating and splenic CD4^+^CXCR5^−^FOXP3^+^ T cells. Labels in plots indicate selected downregulated genes (blue symbols) and upregulated genes (red symbols). (**E**) Bubble chart showed the top 20 differentially expressed Gene Ontology (GO) terms in molecular functions of CD4^+^CXCR5^−^FOXP3^+^ T cells in two groups. Wilcoxon signed-rank test. *p < 0.05, **p < 0.01, ***p < 0.001

Since FOXP3^+^Treg cells residing in peripheral tissues have been shown to display unique functions and gene products relative to their lymphoid tissue counterparts [12], we investigated whether and how the CXCR5 expression influences the FOXP3^+^Treg cells in the peripheral blood and human secondary lymphoid tissue. We first compared the profile of circulating CD4^+^CXCR5^−^FOXP3^+^ T cells and CD4^+^CXCR5^+^FOXP3^+^ T cells. A higher frequency of CXCR5^−^FOXP3^+^ cells was observed relative to CXCR5^+^FOXP3^+^ cells (Additional file 1: Fig. S1 and Additional file 2: Fig. S2A). In addition, CXCR5^−^FOXP3^+^ cells showed reduced ki67 expression and proportion of central memory cells (T_CM_), elevated proportion of effector memory cells (T_EM_), suggesting that CXCR5^−^FOXP3^+^ cells possessed reduced proliferation capacity and enhanced effector function (Additional file 2: Fig. S2B and S2C). Moreover, we found a notable down-regulation of Tim3, IL-10, TGF-β, and elevated granzyme B production in CXCR5^−^FOXP3^+^ cells (Additional file 2: Fig. S2D); there was no significant difference in PD-1, CTLA4, TIGIT, GITR, CD39, CD73, and CD25 expression, suggesting distinct effector function and pathway in CXCR5^−^FOXP3^+^ cells and CXCR5^+^FOXP3^+^ cells. We then compared the features of CD4^+^CXCR5^−^FOXP3^+^ T cells between the peripheral blood and spleen. A higher frequency of CD4^+^CXCR5^−^FOXP3^+^ T cells was observed in the blood than in the spleen (Fig. 1A). Next, we quantified the ki67 expression, a nuclear protein correlated with cellular proliferation. Circulating CD4^+^CXCR5^−^FOXP3^+^ T cells expressed higher levels of ki67 than the spleen (Fig. 1B). In addition, circulating CD4^+^CXCR5^−^FOXP3^+^ T cells showed a lower proportion of T_EM_ cells, a more significant fraction of T_CM_ cells and naïve cells (Fig. 1B), indicating circulating CD4^+^CXCR5^−^FOXP3^+^ T cells exhibited an enhanced proliferation capacity. Of note, we found a down-regulation of PD-1, Tim3, TIGIT, GITR, CD39, and ICOS of CD4^+^CXCR5^−^FOXP3^+^ T cells in the blood and a notable increased CD25 and TGF-β expression. Still, comparable CTLA4, CD73, and IL-10 levels were observed (Fig. 1C), indicating distinct suppressive features between circulating and splenic CD4^+^CXCR5^−^FOXP3^+^ T cells.

To further compare the transcriptional programs of circulating and splenic CD4^+^CXCR5^−^FOXP3^+^ T cells, we analyzed scRNA-seq (Additional file 3: Fig. S3). It demonstrated that splenic CD4^+^CXCR5^−^FOXP3^+^ T cells had 78 up-regulated genes and 22 down-regulated genes (Fig. 1D). Some transcripts encoding suppressive function-associated molecules were observed in the spleen and blood, such as CTLA4, LGALS3, HSPA1B, and KLF3 [13–16]. Further exploration in gene enrichment consisted of genes differentially expressed in these two populations, we found that some cell growth and activation-related Gene Ontology (GO) terms reached statistical significance (Fig. 1E). Our results provide direct evidence for the distinct inhibitory markers and enhanced proliferation capacity in circulating CD4^+^CXCR5^−^FOXP3^+^ T cells, further suggesting that they may be associated with HBV response.

### CD4^+^CXCR5^−^FOXP3^+^ T cells in patients with treatment-naïve chronic HBV infection or with HBV-related hepatic failure have upregulated expression of inhibitory molecules

**Fig. 2:**
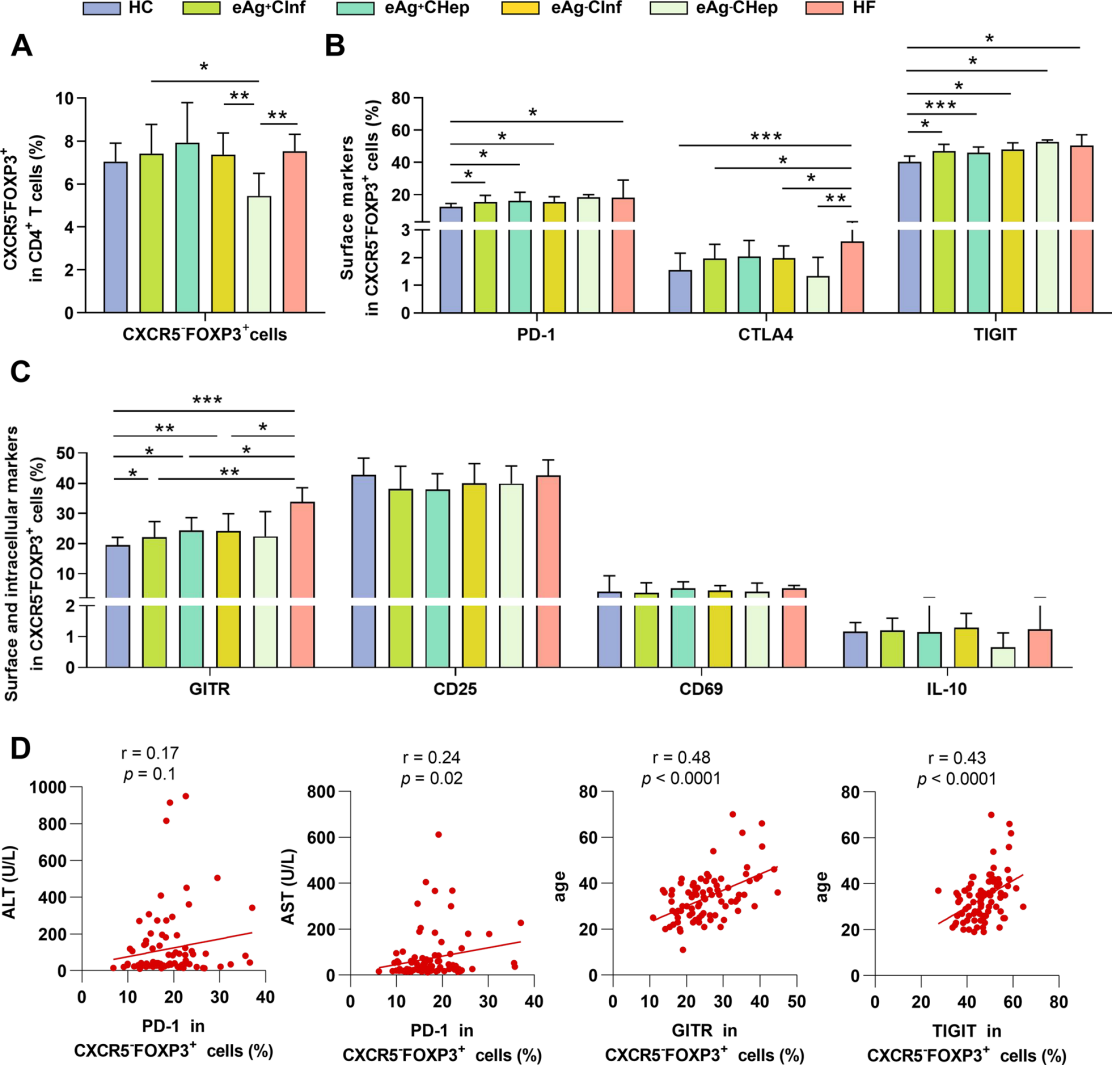
The upregulation of inhibitory features of CD4^+^CXCR5^−^FOXP3^+^ T cells in patients with chronic HBV infection. (**A**) Comparison of frequencies of circulating CD4^+^CXCR5^−^FOXP3^+^ T cells within HC subjects (n = 25) and HBV-infected patients (n = 91). HBV-infected patients, including eAg^+^Clnf (n = 24), eAg^+^CHep (n = 21), eAg^−^Clnf (n = 26), eAg^−^CHep (n = 7) groups, and HBV-related hepatic failure (n = 13). (**B**, **C**) The expression of inhibitory molecules and surface markers in CD4^+^CXCR5^−^FOXP3^+^ T cells among six groups as indicated. (**D**) Correlation between serological index or age and inhibitory markers in CD4^+^CXCR5^−^FOXP3^+^ T cells in HBV-infected patients (n = 91). Mann–Whitney U test, Kruskal–Wallis H test, or Spearman’s rank correlation test. *p < 0.05, **p < 0.01, ***p < 0.001. ALT, alanine aminotransferase; AST, aspartate transaminase; CHB, chronic hepatitis B; eAg^+^CHep, HBeAg-positive chronic hepatitis B; eAg^+^Clnf, HBeAg-positive chronic HBV infection; eAg^−^Chep, HBeAg-negative chronic hepatitis B; eAg^−^Clnf, HBeAg-negative chronic HBV infection; HC, healthy control; HF, hepatic failure

To address the features of circulating CD4^+^CXCR5^−^FOXP3^+^ T cells in chronic HBV infection, we next compared HC subjects, treatment-naïve patients, and patients with HBV-related hepatic failure. Although the frequency of CD4^+^CXCR5^−^FOXP3^+^ T cells in the HBV-infected patients was comparable with HC subjects (Fig. 2A), eAg^−^CHep patients showed notable reduced CD4^+^CXCR5^−^FOXP3^+^ T cell frequency in comparison to eAg^+^CInf, eAg^−^CInf, and hepatic failure patients. We next quantified the inhibitory features expression of CD4^+^CXCR5^−^FOXP3^+^ T cells. It revealed that HBV-infected patients exhibited a substantial increase in the PD-1, TIGIT, and GITR expression compared with HC subjects (Fig. 2B, C). Of note, the levels of CTLA4 and GITR were significantly upregulated in patients with HBV-related hepatic failure. Further analysis of clinical characteristics correlation showed that the PD-1 level on CD4^+^CXCR5^−^FOXP3^+^ T cells was associated positively with ALT and AST (Fig. 2D). Importantly, we found that the expression of GITR and TIGIT on CD4^+^CXCR5^−^FOXP3^+^ T cells was positively correlated with age. Taken together, our results prove the upregulation of inhibitory markers of CD4^+^CXCR5^−^FOXP3^+^ T cells in chronic HBV infection and HBV-related liver failure, further suggesting that they may be associated with dysregulated anti-HBV immune response.

### HBV antigens induce the elevated inhibitory features of CD4^+^CXCR5^−^FOXP3^+^ T cells

**Fig. 3:**
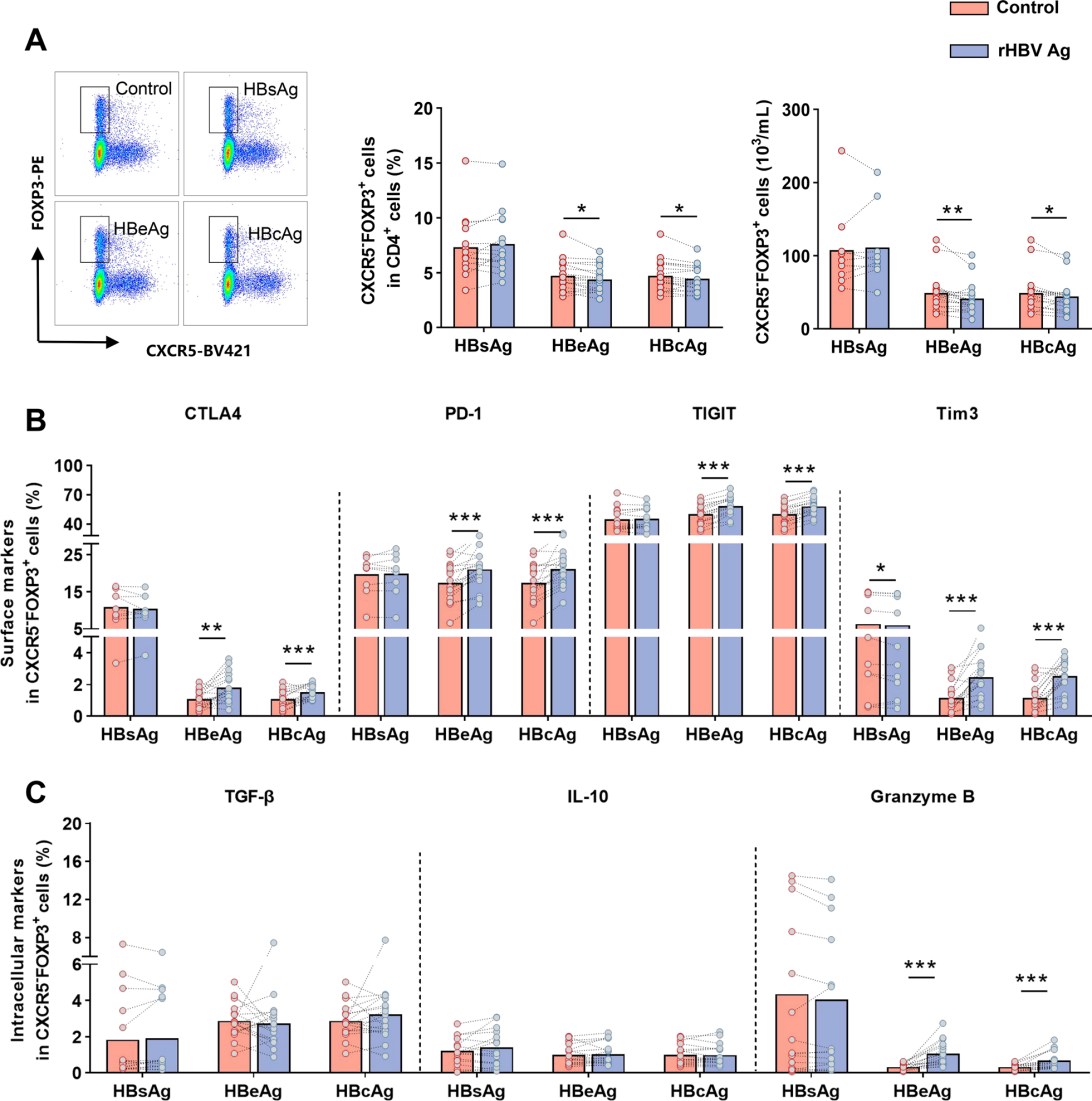
HBV antigens induce the elevated inhibitory molecules of CD4^+^CXCR5^−^FOXP3^+^ T cells. (**A**) The gating strategy for CXCR5^−^FOXP3^+^ T cells in CD4^+^T cells of control, HBsAg, HBeAg, and HBcAg groups (left), and the frequency of circulating CD4^+^CXCR5^−^FOXP3^+^ T cells between control and corresponding HBsAg (n = 15), HBeAg (n = 18), and HBcAg (n = 18) groups. (**B**) Histogram showing the frequencies of surface markers (CTLA4, PD-1, TIGIT, Tim3) in CD4^+^CXCR5^−^FOXP3^+^ T cells with different rHBV Ag or control stimulation. (**C**) Comparison of the frequencies of intracellular molecules (TGF-β, IL-10, and Granzyme B) in CD4^+^CXCR5^−^FOXP3^+^ T cells in the three paired groups, respectively. Wilcoxon signed-rank test. *p < 0.05, **p < 0.01, ***p < 0.001. rHBV Ag, recombination HBV antigen

Since CD4^+^CXCR5^−^FOXP3^+^ T cells exhibited a difference in several phases of HBV infection, we determined whether HBV antigens are directly responsible for this population. We analyzed the effect of recombinant HBsAg (rHBsAg), recombinant HBeAg (rHBeAg), and recombinant HBcAg (rHBcAg) on CD4^+^CXCR5^−^FOXP3^+^ T cells in vitro. It was observed that rHBeAg and rHBcAg induced the minor decline of CD4^+^CXCR5^−^FOXP3^+^ T cells (Fig. 3A), suggesting HBV antigens had a negligible impact on CD4^+^CXCR5^−^FOXP3^+^ T cell frequency. Nevertheless, within the CXCR5^−^FOXP3^+^ T population, the expressions of inhibitory markers (PD-1, CTLA4, TIGIT, Tim3, and Granzyme B) were significantly up-regulated after being stimulated by rHBeAg and rHBcAg, rather than rHBsAg (Fig. 3B, C). In contrast, no significant changes in the expression of IL-10 and TGF-β were observed, implying that HBV antigens influenced the elevated suppressive features of CD4^+^CXCR5^−^FOXP3^+^ T in the HBV infection.

### Decreased circulating CTLA4^+^CD4^+^CXCR5^−^FOXP3^+^ T cells are associated with favorable response in CHB patients with antiviral therapy

**Fig. 4:**
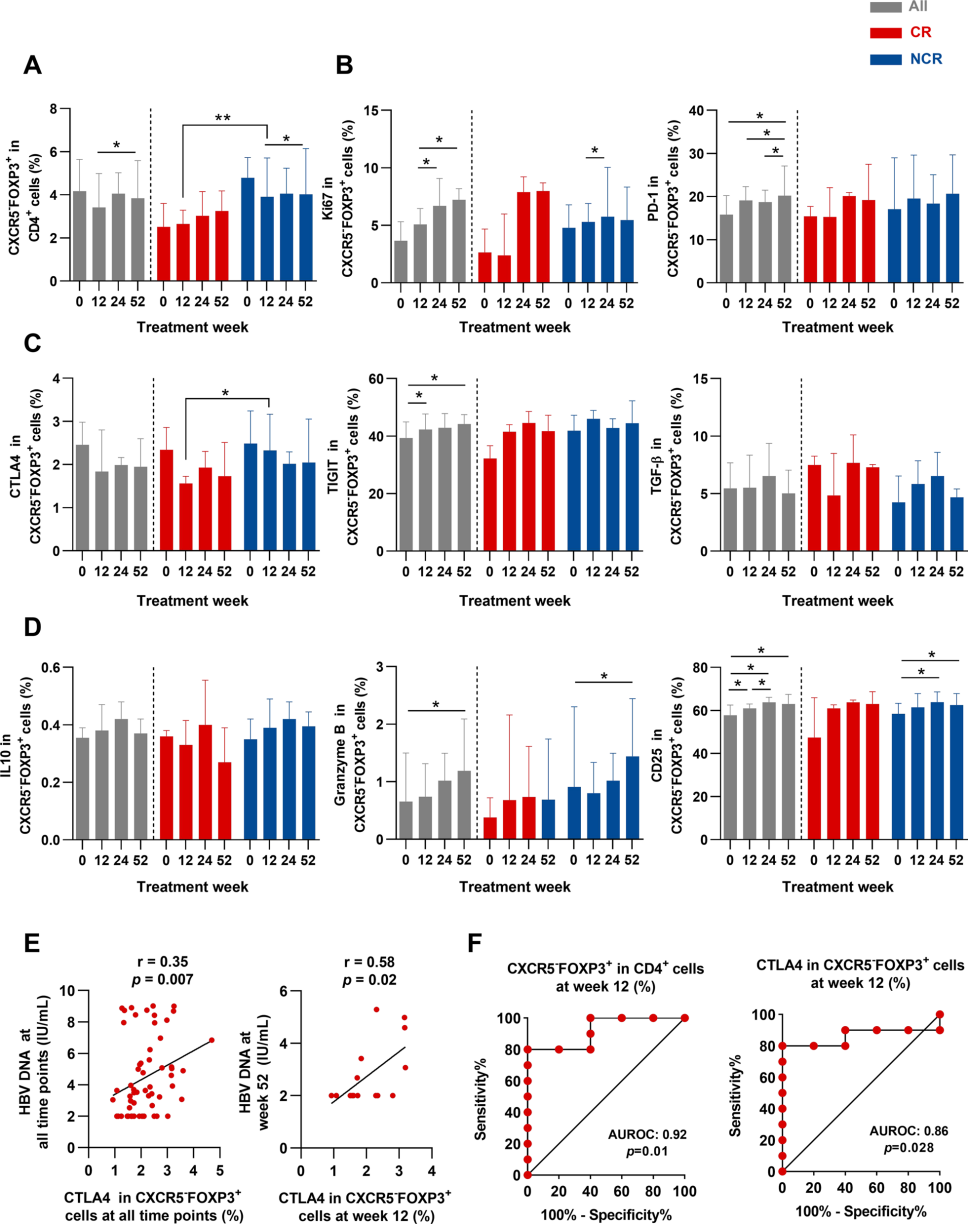
The low frequency of circulating CTLA4^+^CD4^+^CXCR5^−^FOXP3^+^ T cells is associated with virological response in CHB patients. (**A**) The frequency of circulating CD4^+^CXCR5^−^FOXP3^+^ T cells among all subjects (grey, n = 15), CR (red, n = 5), and NCR (blue, n = 10) groups at weeks 0, 12, 24, and 52. (**B**, **C**, **D**) The production of ki67 and indicated molecules in CD4^+^CXCR5^−^FOXP3^+^ T cells among three groups at indicated time points. (**E**) Correlation analysis between serum HBV DNA and the frequency of CTLA4^+^CD4^+^CXCR5^−^FOXP3^+^ T cells at all time points (left), and serum levels of HBV DNA at week 52 and the frequency of CTLA4^+^CD4^+^CXCR5^−^FOXP3^+^ T cells at week 12 (right). (**F**) ROC curves of the frequencies of CD4^+^CXCR5^−^FOXP3^+^ T cells and CTLA4 at week 12 to predict Telbividine-treatment response. Mann–Whitney U test or Wilcoxon signed-rank test. *p < 0.05, **p < 0.01, ***p < 0.001. AUROC, the area under the receiver operating characteristic curve; CR, complete response; NCR, non-complete response; ROC, receiver operating characteristic

We next investigated the relationship between CD4^+^CXCR5^−^FOXP3^+^ T cells and antiviral treatment response. It was observed that antiviral therapy resulted in a significantly elevated frequency of CD4^+^CXCR5^−^FOXP3^+^ T cells with increased ki67, PD-1, TIGIT, Granzyme B, and CD25 expression at 52 weeks of treatment in all patients (Fig. 4A–D). To determine the role of CD4^+^CXCR5^−^FOXP3^+^ T cells in anti-HBV treatment response, patients were divided into CR and NCR groups. Interestingly, the frequency of CD4^+^CXCR5^−^FOXP3^+^ T cells was significantly lower in CR patients at week 12 than in NCR patients (Fig. 4A). As expected, CR patients exhibited a substantial decrease in CTLA4 at week 12 compared to NCR patients (Fig. 4C), suggesting that the level of CTLA4 was associated with anti-HBV treatment response. Further exploration of clinical data showed a positive correlation between the levels of HBV DNA and the expression of CTLA4 at all time points of treatment (Fig. 4E). Notably, the levels of HBV DNA at week 52 showed a positive correlation with the CTLA4^+^CD4^+^CXCR5^−^FOXP3^+^ T cells at week 12 (Fig. 4E).

We further sought to analyze the predictive value of CD4^+^CXCR5^−^FOXP3^+^ T cells, and a receiver operating characteristic (ROC) curve was generated. The area under the curve (AUC) for CD4^+^CXCR5^−^FOXP3^+^ T cells and CTLA4 expression at week 12 indicated that they had a great predictive value for NCR (Fig. 4F). The optimal cut-off value was assessed according to Youden’s index. The optimal cut-off value of CD4^+^CXCR5^−^FOXP3^+^ T cells was 3.395, with a sensitivity of 0.8 and a specificity of 1 (*p* = 0.01). In addition, a cut-off value of CTLA4 expression in CD4^+^CXCR5^−^FOXP3^+^ T cells was 1.785, associated with the best compromise for sensitivity and specificity, respectively at 0.8 and 1 (*p* = 0.03). These data demonstrated that the high frequency of CTLA4^+^CD4^+^CXCR5^−^FOXP3^+^ T cells at 12 weeks of therapy predicted unfavorable outcome in chronic hepatitis B and suggested that this population may contribute to HBV persistence.

## Discussions

In this study, by using a series of samples from HBV-infected patients and a longitudinal cohort of CHB patients with NA treatment, we showed that patients with treatment-naïve chronic HBV infection or with HBV-related hepatic failure have an upregulation of immune-suppressive features (PD-1, CTLA4, and GITR) in circulating CD4^+^CXCR5^−^FOXP3^+^ T cells in response to HBV antigen stimulation. Notably, the frequency of circulating CTLA4^+^CD4^+^CXCR5^−^FOXP3^+^ T cells is associated with virological response in patients undergoing antiviral therapy. These findings reveal previously unappreciated insights into the critical contribution of CTLA4^+^CD4^+^CXCR5^−^FOXP3^+^ T cells to unfavorable outcome of anti-HBV treatment.

The natural history of chronic HBV infection has been classified into several clinical phases involving dynamic cooperation between the virus and the host immune responses [17]. Host establishes and maintains immunological tolerance to viral replication in the HBeAg-positive chronic HBV infection phase, characterized by increased Treg and exhausted T cells in the liver [18]. Similarly, our findings showed that the CD4^+^CXCR5^−^FOXP3^+^ T cells in the peripheral blood had upregulation of immune-suppressive features, suggesting the coexisting inhibitory effects in the peripheral blood and liver account for HBV persistence. Of note, inhibitory features of CD4^+^CXCR5^−^FOXP3^+^ T cells were closely associated with poor outcomes (hepatic failure). Therefore, it might be essential to determine the influencing factors on inhibitory effects. A previous study has reported that IL-10-secreting cells were enriched in CD4^+^CD25^+^ cells in response to HBcAg [11]. Nevertheless, our study revealed the distinct elevated inhibitory features (PD-1, CTLA4, TIGIT, Tim3, and Granzyme B) of CD4^+^CXCR5^−^FOXP3^+^ T cells in response to HBeAg and HBcAg stimulation (Fig. 3), which identify the unique regulation of CD4^+^CXCR5^−^FOXP3^+^ T cells in HBV infection. In addition, we found that age was positively correlated with immune-suppressive molecules on CD4^+^CXCR5^−^FOXP3^+^ T cells (Fig. 2D). This finding indicates that age is an essential factor that influences CD4^+^CXCR5^−^FOXP3^+^ T cells. Indeed, a new perspective regarding age, in other words, the duration of HBV exposure, impacts immune response, which has been proposed by previous research groups [19]. This finding also indicates that young CHB patients should be considered the optimal candidates for anti-HBV treatment.

The role of CD4^+^CXCR5^−^FOXP3^+^ T cells in CHB patients undergoing antiviral treatment has not yet been thoroughly investigated. Data from our longitudinal clinical cohort revealed that antiviral therapy significantly induced CD4^+^CXCR5^−^FOXP3^+^ T cell proliferation and inhibitory features. It was explained by assuming that immune-mediated liver damage was alleviated after treatment followed by increased CD4^+^CXCR5^−^FOXP3^+^ T cells, supported by previous studies [20]. Interestingly, a striking observation from our study was that the low frequency of CTLA4^+^CD4^+^CXCR5^−^FOXP3^+^ T cells after 12 weeks of antiviral therapy predicts HBeAg seroconversion in CHB. It was reported that increased serum IL-21 levels at 12 weeks of treatment benefit HBeAg seroconversion [21, 22]. We speculated that high IL-21 levels restrained CD4^+^CXCR5^−^FOXP3^+^ T cells and their inhibitory features, which might favor the achievement of HBV antigen seroconversion. Nevertheless, due to the limitations of available purified CD4^+^CXCR5^−^FOXP3^+^ T cells in the blood of CHB patients, we have not investigated the function of CD4^+^CXCR5^−^FOXP3^+^ T cells and the mechanisms underlying this effect, which will be the focus of our future studies.

## Conclusions

In summary, we herein described a specialized population of CD4^+^CXCR5^−^FOXP3^+^ T cells with an upregulation of immune-suppressive molecules in HBV-infected patients. Notably, this population with CTLA4 expression at 12 weeks of treatment can predict clinical outcome in CHB. Thus, novel therapeutic strategies against HBV infection could include specific agents targeting CD4^+^CXCR5^−^FOXP3^+^ T cell-mediated immune regulation.

## Methods

### Patients

One hundred and six treatment-naïve patients with chronic HBV infection and twenty-five healthy controls (HCs) were recruited from real-life clinical practice (Additional file 4: Table  S1). According to the European Association for the Study of the Liver (EASL) 2017 clinical practice guideline [23], patients were divided into 4 groups: hepatitis B e antigen-positive chronic infection (HBeAg^+^CInf), HBeAg-positive chronic hepatitis (HBeAg^+^CHep), hepatitis B e antigen-negative chronic infection (HBeAg^−^CInf), and HBeAg-negative chronic hepatitis (HBeAg^−^CHep). Thirteen patients with HBV-related hepatic failure were recruited from real-life clinical practice (Additional file 5: Table S2). Additionally, fifteen HBeAg-positive CHB patients who participated in a clinical trial of telbivudine (number: CLDT600ACN07T) for 52 weeks were longitudinally studied (Additional file 6: Table S3). These subjects were classified into either a complete response (CR, n = 5) group, if they had undergone HBeAg seroconversion and achieved serum HBV DNA levels < 300 copies/ml at week 52, or a non-complete response (NCR, n = 10) for other subjects. Patients suffering from autoimmune diseases, other severe or active diseases, or coinfected with HAV, HCV, HDV, HEV, and HIV were in the exclusion criteria. In addition, splenic tissues were obtained from 11 patients who underwent splenectomy due to HBV-related liver cirrhosis-induced hypersplenism (Additional file 7: Table S4). All individuals were recruited at Nanfang Hospital (Guangzhou, China). Following the Declaration of Helsinki, the Ethical Committee of Nanfang Hospital approved this study and written informed consent was obtained from all participants.

### Cell surface and intracellular cytokine staining (ICS)

Peripheral blood mononuclear cells (PBMCs) from humans and mice were obtained by Ficoll-Hypaque density gradient centrifugation, and then used directly or cryopreserved. Preparation of intrahepatic and splenic lymphocytes from mice was performed according to the procedure described previously [24, 25]. Live cells were identified using Live/Dead staining (Thermo Fisher Scientific, Waltham, MA, USA) at room temperature for 10 min. For phenotype analysis, cells were stained with fluorescence phenotype antibodies at 4℃ for 30 min. For intracellular cytokine staining, cells were fixed and permeabilized with Cytofix/Cytoperm kit (BD Bioscience) or Transcription Factor Buffer Set (BD Bioscience) after phenotype staining and then incubated with corresponding intracellular cytokine antibodies for another 30 min. To assess the function of CD4^+^CXCR5^−^FOXP3^+^ T cells, PBMCs or lymphocytes were stimulated as follows: human cells were cultured with IL-2 (10 ng/mL), anti-CD3/CD28 (5 μg/mL) for 3 days, and Brefeldin A (10 μg/mL) in the last 6 h, mouse cells were stimulated with anti-CD3/CD28 (1 μg/mL), PMA (50 ng/mL), IONO (750 ng/mL) and Brefeldin A for 6 h. All samples were acquired on BD Canto II or Aria III cytometer (BD Bioscience) and analyzed using FlowJo V10.3 software.

### Cell cultures

PBMCs from HBV-infected patients were cultured in RPMI-1640 complete media containing 10% FBS for 3 days with rHBsAg (5 μg/mL), rHBeAg (5 μg/mL), rHBcAg (5 μg/mL), or isotype control. Then, the levels of CD4^+^CXCR5^−^FOXP3^+^ T cells and their surface molecules were analyzed by flow cytometry. For the intracellular cytokines, PBMCs were stimulated with rHBsAg, rHBeAg, rHBcAg, or isotype control for 3 days, and with anti-CD3/CD28 (5 μg/mL) in the last 6 h in the presence of Brefeldin A (10 μg/mL).

### Single-cell sequencing and transcriptomics

We assayed CD4^+^CD25^+^CD127^−^ cells from paired blood and spleen samples of two donors. For each donor, PBMC and splenic lymphocytes were isolated as described above. Then CD4^+^CD25^+^CD127^−^ cells were purified by FACs sort with BD FACS Aria III, ensuring the cell viability of each sample > 80%. Cellular suspensions were adjusted to 1000 cells/μl and loaded on a 10 × Chromium Controller (10 × Genomics). The Single Cell 3’Protocol produces Illumina-ready sequencing libraries. The raw single-cell RNA sequencing (scRNA-seq) data were aligned, filtered, and normalized using the CellRanger by the quality control criteria: (1) the number of uniquely detected genes was 100–3000 per cell, (2) the number of unique molecular identifiers (UMI) < 10,000/cell and mitochondrial gene expression < 20% and finally approximately 6041 to 7490 cells were obtained. ScRNA-seq data analysis, including normalizing, principal component analysis, differential expression analysis, and Gene Ontology (GO) enrichment analysis were performed using the Seurat software. Raw scRNA-seq data were uploaded to the Genome Sequence Archive (GSA) database (GSA accession no. HRA002273).

### Statistical analysis

Statistical analysis was performed in GraphPad Prism software. The Mann–Whitney U test or the Wilcoxon signed-rank test were used for two groups comparison, while the Kruskal–Wallis H test or the Friedman test were used for multiple group comparison. The Spearman’s rank correlation test was used to determine correlations between variables. All statistical analyses were carried out as two-tail tests and significance levels were defined as *p < 0.05, **p < 0.01, and ***p < 0.001 for all tests.

## Supplementary Information

**Additional file 1. Figure S1.** Representative FACS plots showing the gating strategy and the staining of cell markers.

**Additional file 2. Figure S2.** Identification of CD4^+^CXCR5^-^FOXP3^+^ T cells and CD4^+^CXCR5^+^FOXP3^+^ T cells in peripheral blood.

**Additional file 3. Figure S3.** The transcriptomic box plot and cluster dendrogram across samples between the two groups (circulating and splenic CD4^+^CXCR5^-^FOXP3^+^ T cells).

**Additional file 4. Table S1.** Clinical characteristics of healthy control, treatment-naïve chronically HBV-infected patients.

**Additional file 5. Table S2.** Clinical characteristics of patients with HBV-related hepatic failure.

**Additional file 6. Table S3.** Baseline clinical characteristics of HBeAg-positive CHB patients who received telbivudine treatment in longitudinal cohort.

**Additional file 7. Table S4.** Clinical characteristics of patients who underwent splenectomy due to HBV-related liver cirrhosis-induced hypersplenism.

## Data Availability

The datasets analysed during the current study are available in the Genome Sequence Archive (GSA) database (GSA accession no. HRA002273).

## Abbreviations

ALT: Alanine aminotransferase
AST: Aspartate transaminase
AUC: Area under the curve
AUROC: The area under the receiver operating characteristic curve
CHB: Chronic hepatitis B
CR: Complete response
CTLA4: Cytotoxic T-lymphocyte-associated protein 4
eAg^+^CHep: HBeAg-positive chronic hepatitis B
eAg^+^Clnf: HBeAg-positive chronic HBV infection
eAg^−^CHep: HBeAg-negative chronic hepatitis B
eAg^−^Clnf: HBeAg-negative chronic HBV infection
GITR: Glucocorticoid-induced tumor necrosis factor receptor
HBV: Hepatitis B virus
HC: Healthy control
ICS: Intracellular cytokine staining
IL-21R: Interleukin-21 receptor
IL-21R-KO: Interleukin-21 receptor knockout
LAG-3: Lymphocyte activation gene-3
NAs: Nucleos(t)ide analogues
NCR: Non-complete response
PBMCs: Peripheral blood mononuclear cells
PD-1: Programmed death receptor-1
PEG-IFNα: Pegylated interferon-alfa
rHBcAg: Recombinant HBcAg
rHBeAg: Recombinant HBeAg
rHBsAg: Recombinant HBsAg
rHBV Ag: Recombination HBV antigen
ROC: Receiver operating characteristic
T_CM_: Central memory T
T_EM_: Effector memory T
Tfh: Follicular helper T
Tfr: Follicular regulatory T
Tim3: T cell immunoglobulin domain and mucin 3
Treg: Regulatory T
WT: Wild type
