# Supplementary material for: CTLA4+CD4+CXCR5−FOXP3+ T cells associate with unfavorable outcome in patients with chronic HBV infection
Source: BMC Immunol. 2023 Jan 12;24:3. doi: 10.1186/s12865-022-00537-w (PMC9835316; doi:10.1186/s12865-022-00537-w)
Supplement: Supplementary file 1 — Additional file 1. Figure S1. Representative FACS plots showing the gating strategy and the staining of cell markers. [file 12865_2022_537_MOESM1_ESM.docx]

**Additional file 1**

**Figure S1**


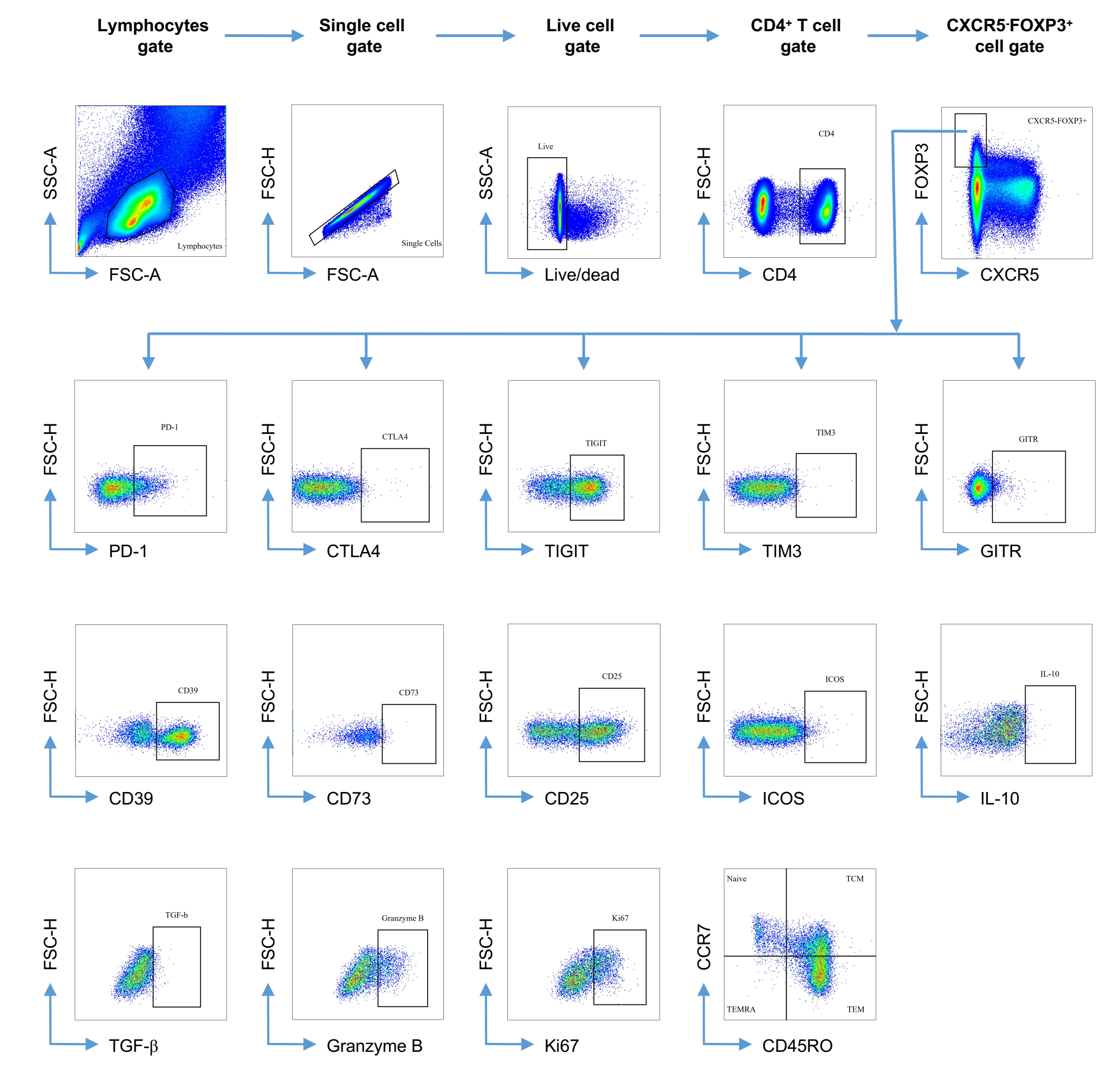


**Fig. S1.** Representative FACS plots showing the gating strategy and the staining of cell markers.
