# Supplementary material for: CTLA4+CD4+CXCR5−FOXP3+ T cells associate with unfavorable outcome in patients with chronic HBV infection
Source: BMC Immunol. 2023 Jan 12;24:3. doi: 10.1186/s12865-022-00537-w (PMC9835316; doi:10.1186/s12865-022-00537-w)
Supplement: Supplementary file 2 — Additional file 2. Figure S2. Identification of CD4+CXCR5-FOXP3+ T cells and CD4+CXCR5+FOXP3+ T cells in peripheral blood. [file 12865_2022_537_MOESM2_ESM.docx]

**Additional file 2**

**Figure S2**


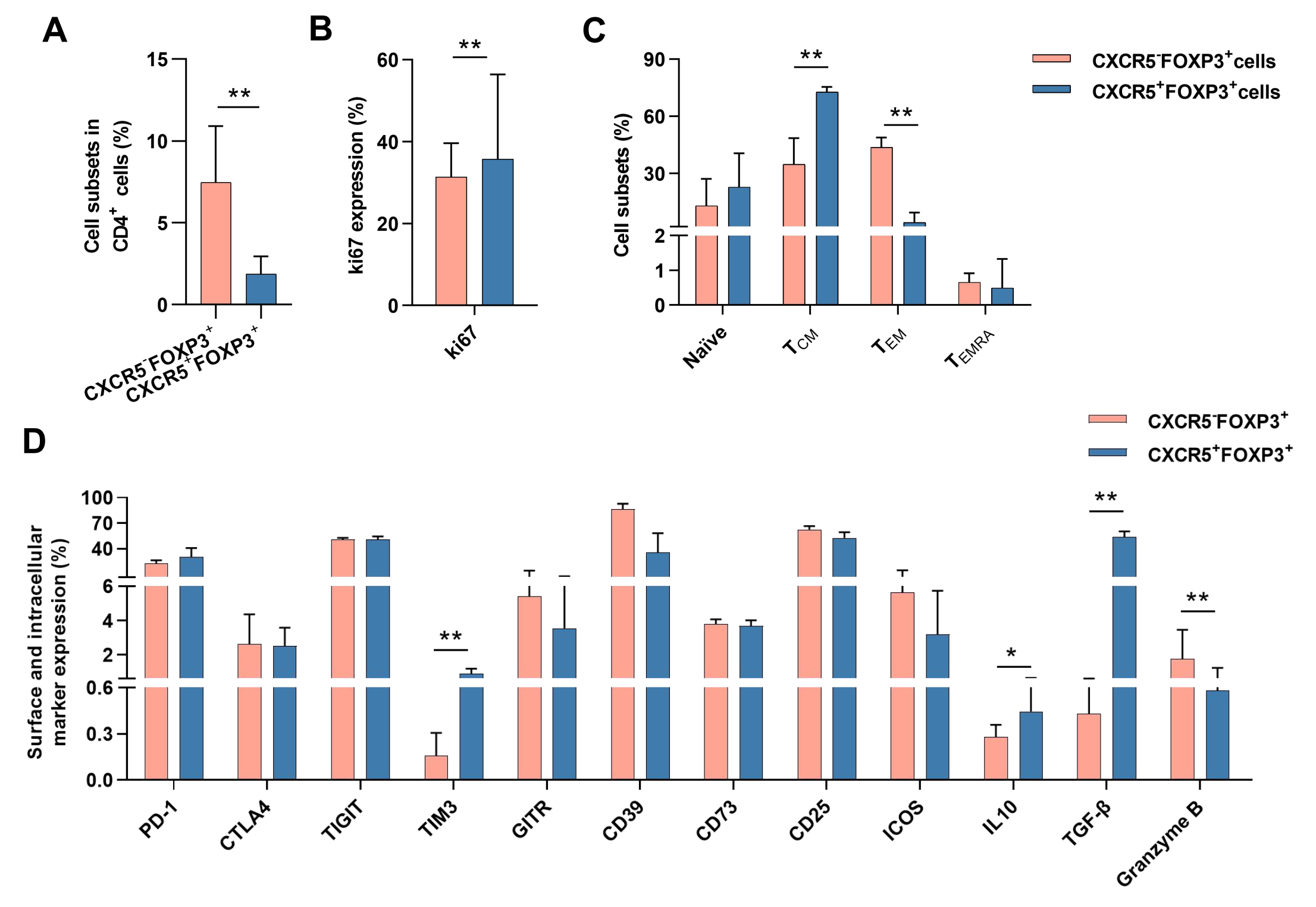


**Fig. S2.** Identification of CD4^+^CXCR5^-^FOXP3^+^ T cells and CD4^+^CXCR5^+^FOXP3^+^ T cells in peripheral blood. (**A**) The frequency of CD4^+^CXCR5^-^FOXP3^+^ T cells and CD4^+^CXCR5^+^FOXP3^+^ T cells in peripheral blood from patients with HBV infection (n=9). Horizontal bars reflect the median. (**B and C**) The percentage of ki67 and T cell subsets (n=9) in CD4^+^CXCR5^-^FOXP3^+^ T cells and CD4^+^CXCR5^+^FOXP3^+^ T cells. T cell subsets were defined using the following gating strategy: naïve T cells (CCR7^+^CD45RO^-^), T_CM_: central memory T cells (CCR7^+^CD45RO^+^), T_EM_: effector memory T cells (CCR7^-^CD45RO^+^), T_EMRA_: CD45RA expressing effector memory T cells (CCR7^-^CD45RO^-^) (**D**) The expression of surface and intracellular cytokines (n=9) in CD4^+^CXCR5^-^FOXP3^+^ T cells and CD4^+^CXCR5^+^FOXP3^+^ T cells. Wilcoxon signed-rank test. **p* <0.05, ***p* <0.01, ****p*<0.001.
