# Supplementary material for: CTLA4+CD4+CXCR5−FOXP3+ T cells associate with unfavorable outcome in patients with chronic HBV infection
Source: BMC Immunol. 2023 Jan 12;24:3. doi: 10.1186/s12865-022-00537-w (PMC9835316; doi:10.1186/s12865-022-00537-w)
Supplement: Supplementary file 3 — Additional file 3. Figure S3. The transcriptomic box plot and cluster dendrogram across samples between the two groups (circulating and splenic CD4+CXCR5-FOXP3+ T cells). [file 12865_2022_537_MOESM3_ESM.docx]

**Additional file 3**

**Figure S3**


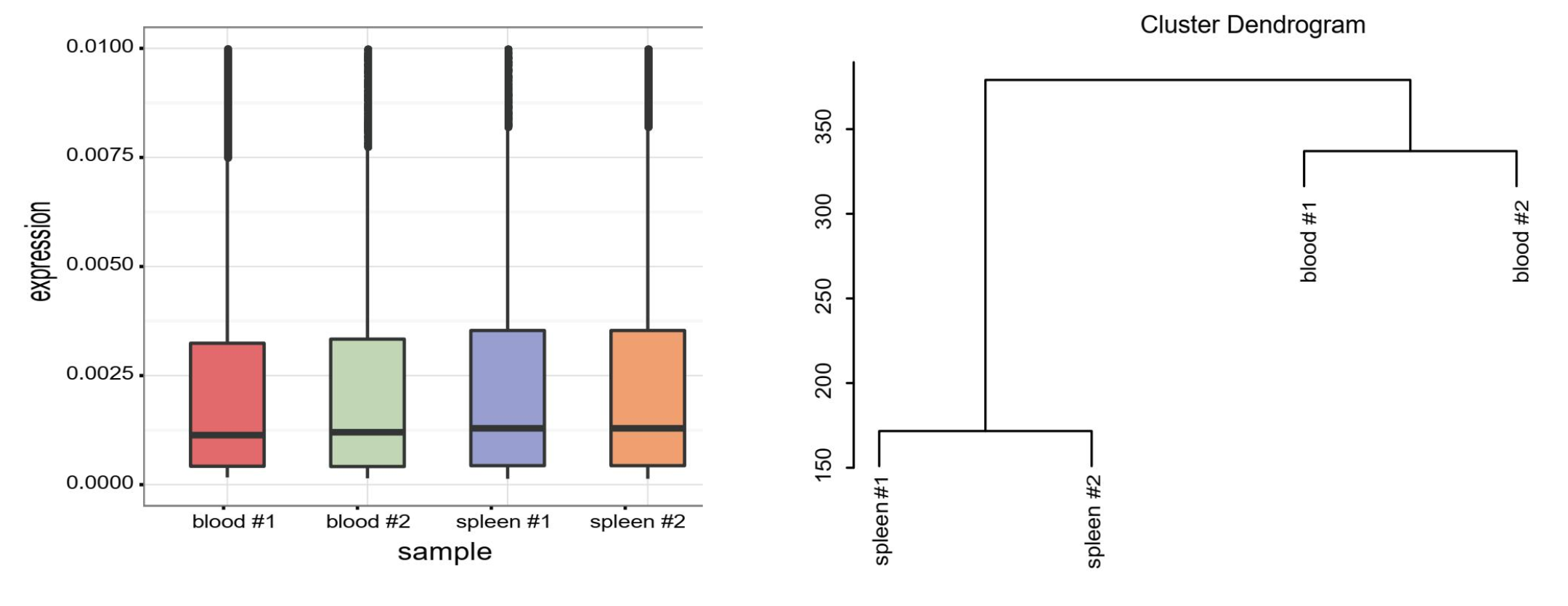


**Fig. S3.** The transcriptomic box plot and cluster dendrogram across samples between the two groups (circulating and splenic CD4^+^CXCR5^-^FOXP3^+^ T cells). The distance parameter for the branch length axis was euclidean and the linkage type was complete.
