# Supplementary material for: CTLA4+CD4+CXCR5−FOXP3+ T cells associate with unfavorable outcome in patients with chronic HBV infection
Source: BMC Immunol. 2023 Jan 12;24:3. doi: 10.1186/s12865-022-00537-w (PMC9835316; doi:10.1186/s12865-022-00537-w)
Supplement: Supplementary file 4 — Additional file 4. Table S1. Clinical characteristics of healthy control, treatment-naïve chronically HBV-infected patients. [file 12865_2022_537_MOESM4_ESM.docx]

**Additional file 4**

**Table S1. Clinical characteristics of healthy control, treatment-naïve chronically HBV-infected patients.**

| Group | HC | eAg+CInf | eAg+CHep | eAg-CInf | eAg-CHep | Ungrouped |
| --- | --- | --- | --- | --- | --- | --- |
| Number | 25 | 31 | 28 | 30 | 7 | 10 |
| Gender (male/female) | 10/14 | 18/13 | 21/7 | 28/2 | 5/2 | 4/6 |
| Age (years) * | 25(19-41) | 26.5(19-47) | 30(21-42) | 35.5(19-54) | 40.5(39-42) | 29(22-56) |
| ALT (IU/L) * | 19(15-74) | 31(11-43) | 136.5(84-505) | 25(13-56) | 119(86-950) | 46.5(20-64) |
| HBV DNA (log_10_IU/mL) * | NA | 7.94(6.91-8.62) | 7.49(5.74-9.03) | 2.42(2-3.77) | 5.83(3.1-7.27) | 6.29(3.2-8.4) |
| HBsAg (IU/mL) * | NA | 36673(894.5-52000) | 18042(1494-52000) | 1108.5(3.6-5976) | 2560.7(283.3-7141.4) | 2791(655.5-52000) |
| HBeAg/anti-HBe | NA | 31/0 | 27/0 | 0/28 | 0/6 | 10/4 |

*Data are shown as median (range); ALT, alanine aminotransferase; anti-HBe, antibody to hepatitis B e antigen; eAg^+^CHep, HBeAg-positive chronic hepatitis B; eAg^+^Clnf, HBeAg-positive chronic HBV infection; eAg^-^Chep, HBeAg-negative chronic hepatitis B; eAg^-^Clnf, HBeAg-negative chronic HBV infection; HC, healthy control; NA, not available. Fig. 2, available data from 25 HC, 24 eAg^+^Clnf, 21 eAg^+^CHep, 26 eAg^-^Clnf and 7 eAg^-^CHep patients; Fig. 3, available data from 33 chronically HBV-infected patients.
