# Supplementary material for: CTLA4+CD4+CXCR5−FOXP3+ T cells associate with unfavorable outcome in patients with chronic HBV infection
Source: BMC Immunol. 2023 Jan 12;24:3. doi: 10.1186/s12865-022-00537-w (PMC9835316; doi:10.1186/s12865-022-00537-w)
Supplement: Supplementary file 5 — Additional file 5. Table S2. Clinical characteristics of patients with HBV-related hepatic failure. [file 12865_2022_537_MOESM5_ESM.docx]

**Additional file 5**

**Table S2. Clinical characteristics of patients with HBV-related hepatic failure.**

| Group | HBV-related hepatic failure |
| --- | --- |
| Number | 13 |
| Gender (male/female) | 10/3 |
| Age (years) * | 41 (33-70) |
| ALT (IU/L) * | 90 (22-914) |
| HBV DNA (log_10_IU/L) * | 4.32 (2-8.23) |
| HBeAg/anti-HBe | 4/6 |

*Data are shown as median (range); ALT, alanine aminotransferase; anti-HBe, antibody to hepatitis B e antigen; NA, not available. Fig. 2, available data from 13 HBV-related hepatic failure patients.
