# Supplementary material for: CTLA4+CD4+CXCR5−FOXP3+ T cells associate with unfavorable outcome in patients with chronic HBV infection
Source: BMC Immunol. 2023 Jan 12;24:3. doi: 10.1186/s12865-022-00537-w (PMC9835316; doi:10.1186/s12865-022-00537-w)
Supplement: Supplementary file 6 — Additional file 6. Table S3. Baseline clinical characteristics of HBeAg-positive CHB patients who received telbivudine treatment in longitudinal cohort. [file 12865_2022_537_MOESM6_ESM.docx]

**Additional file 6**

**Table S3. Baseline clinical characteristics of HBeAg-positive CHB patients who received telbivudine treatment in longitudinal cohort.**

| Group | CR | NCR |
| --- | --- | --- |
| Number | 5 | 10 |
| Gender (male/female) | 2/3 | 7/3 |
| Age (years) * | 26 (24-35) | 25.5 (21-42) |
| ALT (IU/L) * | 142 (55-244) | 82 (45-649) |
| HBV DNA (log_10_IU/L) * | 7.96 (6.24-8.88) | 8.72 (6.84-9.02) |
| HBeAg/anti-HBe | 5/0 | 10/0 |

*Data are shown as median (range); ALT, alanine aminotransferase; anti-HBe, antibody to hepatitis B e antigen; CR, complete response; NA, not available; NCR, non-complete response. Fig. 5, available data from 5 CR and 10 NCR.
