# Supplementary material for: CTLA4+CD4+CXCR5−FOXP3+ T cells associate with unfavorable outcome in patients with chronic HBV infection
Source: BMC Immunol. 2023 Jan 12;24:3. doi: 10.1186/s12865-022-00537-w (PMC9835316; doi:10.1186/s12865-022-00537-w)
Supplement: Supplementary file 7 — Additional file 7. Table S4. Clinical characteristics of patients who underwent splenectomy due to HBV-related liver cirrhosis-induced hypersplenism. [file 12865_2022_537_MOESM7_ESM.docx]

**Additional file 7**

**Table S4. Clinical characteristics of patients who underwent splenectomy due to HBV-related liver cirrhosis-induced hypersplenism.**

| Group | HBV-infected patients |
| --- | --- |
| Number | 11 |
| Gender (male/female) | 9/2 |
| Age (years) * | 46.5 (31-57) |
| ALT (IU/L) * | 27 (14-229) |
| HBV DNA (log_10_IU/L) * | 2 (2-6.3) |
| HBeAg/anti-HBe | 2/2 |

*Data are shown as median (range); ALT, alanine aminotransferase; anti-HBe, antibody to hepatitis B e antigen; NA, not available. Fig. 1A, 1B, and 1C, available data from 9 HBV-infected patients; Fig. 1D and 1E, available data from 2 HBV-infected patients.
